# Early effects of transcatheter aortic valve implantation and aortic valve replacement on myocardial function and injury: insights from Cardiovascular Magnetic Resonance

**DOI:** 10.1186/1532-429X-16-S1-P285

**Published:** 2014-01-16

**Authors:** Gareth Crouch, Jayme Bennetts, Ajay Sinhal, Phillip J Tully, Darryl Leong, Craig Bradbrook, Amy Swan, Jawad Mazhar, Carmine De Pasquale, Majo Joseph, Robert A Baker, Joseph Selvanayagam

**Affiliations:** 1 Centre for Cardiovascular Magnetic Resonance Research, Flinders University, Bedford Park, South Australia, Australia; 2 Cardiothoracic Surgery, Flinders Medical Centre, Adelaide, South Australia, Australia; 3 Cardiovascular Medicine, Flinders Medical Centre, Adelaide, South Australia, Australia

## Background

To assess the early effects on myocardial function, reversible and irreversible myocardial injury of both transcatheter (TAVI) and open aortic valve replacement (AVR) utilizing cardiovascular magnetic resonance (CMR) and biochemistry. Although recent studies have reported on clinical outcomes, there has been a paucity of mechanistic data on the effect of TAVI on ventricular function and irreversible myocardial injury.

## Methods

We conducted a prospective comparative study of 47 patients (26 TAVI, 21 AVR) with severe aortic stenosis and normal or mildly impaired ventricular function undergoing either TAVI or high risk AVR. CMR (for LV/RV function, LV mass, oedema, irreversible injury, and aortic valve regurgitation) was carried out pre- and early post-operatively ( < 2 weeks). Biochemistry, high-sensitivity troponin and Pro B-type natriuretic peptide, and transthoracic echocardiography were also performed.

## Results

Groups were similar with respect to STS Score (TAVI 7.7 vs AVR 5.9 p = 0.11) and comorbidities, however the TAVI group were older (84.6 yrs ± 6 vs 79.6 yrs ± 4, p < 0.001). Preoperative left ventricular (LVEF: TAVI 69% ± 13 vs AVR 73% ± 10 p = 0.10) and right ventricular ejection fractions (RVEF: TAVI 61% ± 11 vs AVR 59% ± 8 p = 0.5) were similar. Post-operative LVEF was preserved in both groups. In contrast, a decline in RVEF was notably more significant in the TAVI group (61% to 54% vs 59% to 58%, p = 0.01). Further analysis showed a trend (p = 0.08) association between the degree of post-procedure aortic regurgitation and RV dysfunction. There were no differences in the rate of reversible (T2)irreversible (LGE) injury.

## Conclusions

There was no significant difference in early left ventricular systolic function between open and transcatheter techniques, matched by similar myocardial injury rates. While RV systolic function was preserved in the AVR group, it was significantly impaired early after TAVI, correlating with the degree of para-valvular aortic regurgitation.

## Funding

Funding was provided in the form of unencumbered research grants from St Jude Medical and Edwards Lifesciences.

